# Clinical and imaging features of women with polygenic partial lipodystrophy: a case series

**DOI:** 10.1038/s41387-024-00260-y

**Published:** 2024-02-06

**Authors:** Wann Jia Loh, Jadegoud Yaligar, Amanda J. Hooper, Suresh Anand Sadananthan, Yeshe Kway, Su Chi Lim, Gerald.F. Watts, Sambasivam Sendhil Velan, Melvin Khee Shing Leow, Joan Khoo

**Affiliations:** 1 Department of Endocrinology, Changi General Hospital, Singapore, Singapore; 2 Duke-NUS Medical School, Singapore, Singapore; 3 Singapore Institute for Clinical Sciences, Agency for Science Technology and Research, Singapore, Singapore; 4 Department of Biochemistry, Pathwest and Fiona Stanley Hospital Network, Perth, Australia; 5 School of Medicine, University of Western Australia, Perth, Australia; 6 Departments of Medicine and Physiology, NUS Yong Loo School of Medicine, NUS, Singapore, Singapore; 7 Diabetes Centre, Admiralty Medical Centre, Singapore, Singapore; 8 Department of Cardiology and Internal Medicine, Royal Perth Hospital, Perth, Australia; 9 Department of Endocrinology, Tan Tock Seng Hospital, Singapore, Singapore; 10 LKC School of Medicine, NTU, Singapore, Singapore

**Keywords:** Type 2 diabetes, Metabolic syndrome

## Abstract

**Background:**

Familial partial lipodystrophy (FPLD) is an inherited disorder of white adipose tissue that causes premature cardiometabolic disease. There is no clear diagnostic criteria for FPLD, and this may explain the under-detection of this condition.

**Aim:**

This pilot study aimed to describe the clinical features of women with FPLD and to explore the value of adipose tissue measurements that could be useful in diagnosis.

**Methods:**

In 8 women with FPLD and 4 controls, skinfold measurements, DXA and whole-body MRI were undertaken.

**Results:**

Whole genome sequencing was negative for monogenic metabolic causes, but polygenic scores for partial lipodystrophy were elevated in keeping with FPLD type 1. The mean age of diagnosis of DM was 31 years in the FPLD group. Compared with controls, the FPLD group had increased HOMA-IR (10.3 vs 2.9, *p* = 0.028) and lower mean thigh skinfold thickness (19.5 mm vs 48.2 mm, *p* = 0.008). The FPLD group had lower percentage of leg fat and an increased ratio of trunk to leg fat percentage on DXA. By MRI, the FPLD group had decreased subcutaneous adipose tissue (SAT) volume in the femoral and calf regions (*p* < 0.01); abdominal SAT, visceral adipose tissue, and femoral and calf muscle volumes were not different from controls.

**Conclusion:**

Women with FPLD1 in Singapore have significant loss of adipose but not muscle tissue in lower limbs and have early onset of diabetes. Reduced thigh skinfold, and increased ratio of trunk to leg fat percentage on DXA are potentially clinically useful markers to identify FPLD1.

Lipodystrophies are a group of disorders characterised by the loss of adipose tissue from the subcutaneous compartment and are classified according to whether the distribution of loss of subcutaneous adipose tissue is considered to be familial or acquired, and partial or generalised [1]. Familial partial lipodystrophy (FPLD) is an under-recognised condition characterised by increased genetic predisposition to abnormalities in white adipose tissue function, quantity, and distribution [2–4]. The under-diagnosis of FPLD is potentially detrimental to the patient, and early treatment may prevent sequelae. These include early onset severe insulin resistance, type 2 diabetes mellitus (T2DM), steatohepatitis, hypertriglyceridemia, hypertension, ovarian hyperandrogenism in women, mood disorders, and premature coronary artery disease and death [3–8].

One of the main limiting factors contributing to the under-recognition of FPLD is the lack of clear diagnostic criteria to diagnose FPLD in clinical practice. A few small studies in FPLD have investigated potential clinical tools, such as skinfold thickness measurement, and imaging by dual-energy x-ray absorptiometry (DXA) [5, 9, 10] and whole-body magnetic resonance imaging (MRI) [7, 11–13]. Meral et al. proposed that fat shadows from DXA body composition scans could help to diagnose certain subtypes of lipodystrophy syndromes, such as FPLD1 (otherwise called Köbberling variety) and FPLD2 (otherwise called Dunnigan variety) [9]. However, there is currently no clear consensus on the diagnostic criteria based on skinfold thickness, DXA, or MRI imaging [1, 14].

This pilot study aimed to characterise the features of Singaporean women with the polygenic form of FPLD and to explore the value of measurement of fat distribution in making a phenotypic diagnosis.

## Research design and methods

### Participants

Eight patients with FPLD managed at the endocrinology clinic of Changi General Hospital were studied. They had been diagnosed based on the presence of preferential and symmetrical fat loss of the legs, with normal or increased distribution of fat on the face, neck and trunk, in the presence of a positive family history of a first-degree family member with a similar physical appearance [1, 14]. All patients with FPLD had previously undergone extensive history taking and endocrine testing to rule out diagnosis of endogenous and exogenous Cushing’s syndrome. None of the participants had human immunodeficiency virus infection, history of anti-retroviral therapy or cancer. The four female controls did not have lipodystrophy and were age, ethnicity and BMI-matched, were recruited from endocrinology clinics. Diabetes mellitus (DM) was identified as fasting glucose ≥7 mmol/L, HbA1c ≥ 6.5% or by use of anti-hyperglycaemic medications. Hospital electronic records and patient interviews were used to confirm diagnoses of comorbidities including hypertension, ischaemic heart disease, hypertriglyceridemia, polycystic ovary syndrome (PCOS), and chronic renal disease. This study was approved by the hospital’s ethics committee. All study participants provided written informed consent.

### Skinfold measurement and bioelectrical impedance analysis

All participants underwent detailed physical examination, measurement of waist, hip and neck circumferences and bioelectrical impedance analysis (BIA) using a Tanita body composition analyser. A 7-site skinfold measurement using Harpenden calipers was performed encompassing triceps, biceps, subscapular, suprailiac (otherwise known as supraspinale), abdomen, thigh, and calf of the right side of the body. The anatomy landmarks were as described in the international standards for anthropometric assessment, recommended by the International Society for the Advancement of Kinanthropometry [15]. The mean of two skinfold readings for each of the seven sites was calculated. To minimise inter-observer variability, the measurements were performed by either of the two trained personnel. The sum of 7-site skinfolds was calculated. The calculated skinfold ratios were subscapular to thigh ratio, subscapular to calf ratio, suprailiac to thigh ratio, and suprailiac to calf ratio.

### Laboratory tests

Fasting venous blood samples were collected for biochemical and genetic testing. Serum total cholesterol, triglyceride, high-density lipoprotein cholesterol (HDL-C) and low-density lipoprotein cholesterol concentrations (LDL-C) were measured using an enzymatic colorimetry Roche Cobas c702 analyzer with inter-assay CVs *<*1.5%. Plasma lipoprotein(a) level [Lp(a)] was measured using molar concentration by particle-enhanced turbidimetric immunoassay with Tina-quant Lipoprotein(a) Gen.2 (Latex) Roche. Serum insulin and C-peptide were measured using an Abbott Alinity analyzer. The homeostasis model assessment index of insulin resistance (HOMA-IR) was calculated from the fasting plasma glucose and insulin concentrations [16]. Adipokines (leptin, total adiponectin, chemerin) were measured using Quantikine^TM^ ELISA kits (R&D Systems, Minneapolis USA) with intra and inter-assay CV ≤ 7%. Genomic DNA was extracted from peripheral blood leukocytes using desalting method.

### Dual-energy X-ray absorptiometry (DXA)

DXA scans were performed using Hologic QDR 4500 A, fan-beam densitometer (Hologic, Inc., Bedford, Massachusetts, software version 8.21) to measure whole body composition. Body composition measures included fat of total body, head, trunk, arms and legs, abdominal visceral adipose tissue (VAT), android and gynoid fat masses and ratio of trunk to leg fat percentage. The variable ratio of trunk to leg fat percentage is automatically derived by Hologic software which reported the variable as ‘%fat trunk to % fat of legs ratio’. The precision estimate (%CV) for fat mass using DXA measurement ranges from 1 to 2%. Long term precision QC monitoring on phantoms for three years on the Hologic densitometer was 0.01%. Detailed descriptions of the DXA, genetics and MRI techniques used are in the Supplementary Table 1.

### MRI

Whole body MRI was performed for all participants using Siemens Prisma 3 T MR scanner except for 1 patient with an implantable cardioverter defibrillator who used Siemens Sola 1.5 T MR scanner. The following variables were quantified using MRI: subcutaneous adipose tissue (SAT) [including deep (DSAT) and superficial (SSAT)], visceral adipose tissue (VAT) [including intraperitoneal (IPAT) and retroperitoneal (RPAT)], paraspinal adipose tissue (PSAT) between L1 and L5 vertebrae [17], thigh and calf SAT, as well as thigh and calf intramuscular adipose tissue (IMAT). The liver and pancreatic fat were determined from the multi-echo Dixon fat-water imaging sequence and quantified as mean proton density fat fraction (PDFF). The intramyocellular (IMCL) and extramyocellular (EMCL) lipids within the soleus muscle were determined using magnetic resonance spectroscopy [18, 19]. Gluteal fat thickness was measured from the pelvic MRI as described in a recent study [20]. Using adipose tissue volume quantified using MRI, the following ratios were calculated: abdominal VAT to abdominal SAT ratio, abdominal VAT to thigh SAT ratio, abdominal VAT to calf SAT ratio, abdominal SAT to thigh SAT ratio and abdominal SAT to calf SAT.

### Genetics

Whole genome sequencing was performed by Beijing Genomics Institute (BGI). Lipodystrophy, obesity, hypertriglyceridemia, diabetes, and insulin resistance genes were analysed for rare variants (gnomAD global and East Asian allele frequency <1%) occurring in coding regions or within 20 nucleotides of exon-intron boundary (Supplementary table 2). Variants were annotated, filtered, and analysed in Alissa Interpret Research (Agilent Technologies) and described using Human Genome Variation Society nomenclature version 20.05. A 53-SNP lipodystrophy polygenic score was calculated as previously described by Lotta et al, by summing the total number of risk alleles [21].

### Statistical analyses

Baseline characteristics for continuous variables were expressed in mean ± standard deviation, while for categorical variables, number (n) and percentages were shown. Fischer’s exact test was used for comparison of categorical variables while Mann-Whitney U test was used for continuous variables. Statistical analysis was performed using Statistical Package of Social Science (SPSS Inc, Chicago, IL, USA), version 16.0 for Windows. A *p*-value less than 0.05 was taken as statistically significant.

## Results

### Characteristics

The clinical characteristics and selected results of patients with FPLD are individually described in Table 1. The clinical characteristics and blood results of FPLD and control groups are presented in Table 2. The mean age of FPLD and control groups were 39 and 49.5 years, and their BMI kg/m^2^ were similar at 31.1 and 33.5 respectively. (Table 2). All 8 patients in the FPLD group had T2DM with a mean disease onset at age 31 years (7 patients diagnosed before age 40 years), whereas only 1 control had T2DM with onset at age 39 years old. Metabolic comorbidities were prevalent in the FPLD group, despite their young age; hypertension (*n* = 5), hypertriglyceridemia (*n* = 4), hypercholesterolemia (*n* = 8), PCOS (*n* = 3), CKD (*n* = 2), and ischaemic heart disease (IHD) (*n* = 5). Among the 4 patients with IHD, one had acute myocardial infarction at 36 years old, and another patient with acute coronary syndrome at 40 years old (Table 1), with the other two young women having plaques present on CT coronary angiogram. One of the patients (patient 3) had young onset diabetes since the age of 25 and had been on dialysis for diabetic nephropathy she was 36 years old. All but one patient with FPLD had DM nephropathy (albuminuria or renal impairment). None of the study participants had elevated total testosterone (all < 2 nmol/L).

**Table 1:** Individual characteristics of 8 female patients with polygenic form of familial partial lipodystrophy (FPLD1).

	Patient 1	Patient 2	Patient 3	Patient 4	Patient 5	Patient 6	Patient 7	Patient 8
BMI	25.6	27.0	29.2	32.2	32.5	33.7	33.9	34.9
Age (yr)	37	40	36	46	52	38	25	43
Ethnicity	Chinese	Malay	Chinese	Chinese	Chinese	Chinese	Malay	Chinese
IHD imaging assessment	Non-calcified plaque in LAD. A few mural plaques in the carotid arteries.	NSTEMI (36yo), coronary stent inserted. Reduced EF 35%	Plaques in LAD (20% stenosis). Reduced EF 40%	No cardiac imaging	No cardiac imaging	No cardiac imaging	High-risk plaques present on CT coronary angiogram	IHD -TVD with acute coronary syndrome (stent inserted) at 40 y
Age of DM (y)	34	34	25	22	50	31	14	31
Initial HbA1c(%)	10.2 Abscess	GDM then T2DM	9.8%, multiple admissions	7.5	7.5	8	16.8% DKA, abscess	8.2
HbA1c at enrolment (%)	8	8.2	9.2	8	6.1	10.2	10.2	7.6
HOMA-IR	1.7	9.68	28.8	9.79	4.77	9.51	9.48	8.54
DM medications	3 OGLD	Basal insulin and 3 OGLD	Basal bolus insulin regimen	Basal bolus insulin & 2 OGLD	3 OGLD	4 OGLD	Insulin pump +metformin	4 OGLD
DM nephropathy	0	GFR 50 ml/min albuminuria	ESRF (dialysis)	albuminuria	albuminuria	albuminuria	albuminuria	albuminuria
Highest recorded TG (mmol/L)	2.07	6.71	>50	2.12	2.16	2.67	5.5	1.96
Pre-treatment LDL (mmol/L)	4.44	4.02	4.5	4.79	5.5 with xanthelasma	3.42	4.6 -7 mmol/L	2.89
Family history of metabolic complication in a parent with similar body shape distribution	Unclear, but no premature IHD	premature IHD and DM.	Died of stroke at age 47	DM and hypertension	Dialysis at 60yo (DM ESRF). Died after septic leg amputation	Died of gallbladder cancer at 50 y	Died of acute myocardial infarction at 59 y	Died of DM and kidney failure at 57 y
Thigh skinfold (mm)	13.5	5.4	23.4	17.3	22.0	13.3	33.6	27.1
Scapular to thigh skinfold ratio	2.32	4.98	1.46	1.99	1.85	2.56	1.28	1.43
Suprailiac to thigh skinfold ratio	2.19	3.7	1.74	1.82	1.37	2.08	1.29	1.11
Body Fat % (DXA)	38.7	36.8	40.2	38.8	46.1	42.1	40.1	48.4
Android to gynoid ratio	1.07	1.55	1.17	1.43	1.14	1.53	1.04	1.25
Fat % trunk to fat % legs ratio	1.22	1.88	1.31	1.57	1.26	1.67	1.09	1.40
% fat trunk/%fat leg percentile	98	99	99	99	98	99	96	99
Trunk/limb fat mass ratio	1.49	2.21	1.58	2.08	1.59	1.88	1.22	1.72
Trunk/limb fat mass ratio percentile	98	99	99	99	98	99	95	99
53-SNP gene score*	57	59	57	65	62	58	67	58
*PNPLA3* rs738409 C > G	CG	CC	CC	GG	CC	CG	CG	CG
Liver PDFF (%)	29.77	4.39	3.24	34.94	15.34	11.59	22.47	11.59
Pancreas PDFF (%)	5.32	6.02	3.92	4.79	1.22	14.88	1.67	14.88

Patients are listed in ascending BMI (kg/m^2^).

*Age of DM* age of diagnosis of type 2 diabetes mellitus (DM) Initial HbA1c: the initial HbA1c at diagnosis of DM.

*DM medications* T2DM medications at study enrolment.

*TG* triglyceride.

*PDFF* proton density fat fraction on MRI.

*GDM* gestational diabetes mellitus.

*OGLD* oral glucose lowering agent.

*EF* ejection fraction.

*ESRF* end stage renal failure.

*DKA* diabetic ketoacidosis.

*GFR* glomerular filtration rate.

*LAD* left anterior descending artery.

*Fat % trunk to fat % legs ratio* percentage of fat in trunk divided by percentage of fat in legs.

*Refer to Lotta et al. [21].

**Table 2:** Clinical characteristics and body measurements of female patients with polygenic form of familial partial lipodystrophy (FPLD) compared with female controls.

	FPLD (*n* = 8)	Controls (*n* = 4)	*P* value
Age (years)	39.6 ± 7.9	49.5 ± 10.5	0.121
Weight (kg)	76.2 ± 9.1	81.2 ± 14.1	0.683
BMI (kg/m^2^)	31.1 ± 3.4	33.5 ± 6.3	0.570
***Comorbidities, n (%)***			
T2DM	8 (100)	1 (25)	0.018
Age of onset of T2DM (years)	31 (23.5, 34)	39	0.241
Young onset T2DM < 40 years old	7 (87.5)	1 (25)	0.889
Hypertension	5 (62)	1 (25)	0.273
Hypertriglyceridemia	4 (50)	1 (25)	0.576
Hypercholesterolemia	8 (100)	3 (73)	0.333
PCOS	3 (37.5)	0	0.491
Diabetes retinopathy	7 (87.5)	0	0.010
Diabetes nephropathy	7 (87.5)	0	0.010
Chronic kidney disease	2 (25)	0	0.515
Ischaemic heart disease	3 (37.5)	0	0.491
***Bloods***			
HbA1c (%)	8.2 ± 1.7	5.9 ± 0.7	0.097
ALT (U/L)	41.0 ± 44.3	56.8 ± 56.9	0.933
Creatinine (µmol/L)	123 ± 182	60 ± 14	0.933
Triglyceride (mmol/L)	2.18 ± 1.05	2.12 ± 1.05	1.000
LDL cholesterol (mmol/L)	3.23 ± 1.35	3.12 ± 0.72	0.683
HDL cholesterol (mmol/L)	1.21 ± 0.44	1.32 ± 0.27	0.351
Lp(a) (nmol/L)	67 ± 63, median: 42.7(20.4,113.8)	15 ± 16, median:8.1(5.6, 24.6)	0.073
C reactive protein (mg/L)	3.7 ± 3.0	3.6 ± 3.2	0.897
Fasting insulin (mIU/L)	27.7 ± 32.5, median: 15.7 (12.9, 25.8)	11.5 ± 4.2, median: 11.9(8.0,14.9)	0.154
Fasting glucose (mmol/L)	10.2 ± 4.7	5.4 ± 1.4	0.032
Fasting C-peptide (pmol/L)	1132 ± 498	950 ± 190	0.461
HOMA-IR	10.3 ± 8.0	2.91 ± 1.54	0.028
Leptin (ng/mL)*	44.6 ± 41.1, median: 30.8(20.7,46.9)	58.0 ± 24.1, median: 67.1(42.5,73.4)	0.040
Adiponectin (µg/mL)*	3.00 ± 2.60	4.45 ± 2.46	0.164
Chemerin (ng/mL)*	126.5 ± 49.4	105.0 ± 45.3	0.648
***Tape measurements***			
Neck circumference (cm)	38.3 ± 3.4	35.8 ± 2.4	0.230
Waist circumference (cm)	103.4 ± 7.5	99.0 ± 14.4	0.461
Hip circumference (cm)	105 ± 9.2	110.8 ± 13.2	0.727
Waist to hip ratio	0.98 ± 0.05	0.89 ± 0.03	0.004
Waist to height ratio	0.66 ± 0.04	0.64 ± 0.10	0.461
***Skinfold measurement***			
Biceps (mm)	16.9 ± 4.7	27.8 ± 16.3	0.368
Triceps (mm)	26.3 ± 7.1	30.5 ± 16.9	0.808
Scapular (mm)	35.4 ± 5.2	39.7 ± 12.1	0.683
Abdominal (mm)	40.8 ± 8.9	41.3 ± 5.52	0.808
Suprailiac (mm)	31.6 ± 7.4	37.3 ± 11.3	0.283
Thigh (mm)	19.5 ± 8.9	48.2 ± 11.5	0.008
Calf (mm)	15.6 ± 6.4	24.6 ± 7.6	0.109
Sum of 7 skinfolds (mm)	186 ± 35	249 ± 66.2	0.109
Subscapular to Thigh ratio	2.23 ± 1.20	0.84 ± 0.22	0.004
Subscapular to Calf ratio (KöB index)	2.65 ± 1.17	1.65 ± 0.39	0.073
Suprailiac to Thigh ratio	1.91 ± 0.82	0.77 ± 0.12	0.004
Suprailiac to Calf ratio	2.28 ± 0.82	1.55 ± 0.41	0.073

Values represented as mean ± standard deviation or number (%). Values with skewed distributions were also presented in median with interquartile ranges.

*BMI* body mass index, *T2DM* type 2 diabetes mellitus, *PCOS* polycystic ovary syndrome, *LDL* low-density lipoprotein concentration, *HDL* high density lipoprotein concentration, *Lp(a)* lipoprotein(a), *HOMA-IR* homeostasis model assessment-estimated insulin resistance, *ALT* alanine transaminase, *HbA1c* haemoglobin A1c; *PCOS* polycystic ovarian syndrome.

*For the adipokines concentration, the mean ± SD shown did not include results from a patient with renal failure on dialysis because renal failure causes elevated adipokines concentration.

When compared with controls, the FPLD group had a higher mean HOMA-IR (10.3 vs 2.91, *p* = 0.028), and mean fasting glucose (10.2 vs 5.4 mmol/L, *p* = 0.032). Lp(a), HbA1c and fasting C-peptide trended higher in the FPLD group compared with controls but were not statistically significant. C-reactive protein was not significantly different between the two groups. Excluding the patient with renal failure on dialysis (patient 3), the mean leptin levels were lower in the FPLD group compared with controls (*p* = 0.04), while adiponectin and chemerin levels were not significantly different. HOMA-IR was positively associated with increased VAT volume (quantified by MRI) on univariable linear regression analysis (*p* = 0.023) and multivariable regression analysis adjusting for age, BMI, diagnosis of FPL (coefficient 0.008 [95% CI 0.002-0.013] *p* = 0.012).

### Genetics

Whole genome sequencing of FPLD patients was negative for monogenic causes of FPLD, obesity, diabetes, severe hypertriglyceridemia, and severe insulin resistance. Rare nonsynonymous variants detected within coding regions of these genes of interest are listed in Supplementary Table 2, however, none were classified as pathogenic or likely pathogenic. The polygenic scores using a 53-SNP polygenic score were elevated at 57 to 67 (Table 1); the polygenic scores were at or above the median of the FPLD1 patients described by Lotta et al. [21]. Carrier status for the *PNPLA3 rs738409* polymorphism, rs738409, which is common (gnomAD minor allele frequency 0.38 in East Asians) and associated with hepatic steatosis is also provided (Table 1) [22]. FPLD patient #4 had the highest liver fat percentage in this FPLD group and was homozygous for rs738409.

### Body adipose tissue (fat) distribution

The FPLD group had an increased waist to hip ratio compared with the control group (0.98 vs 0.89, p = 0.004) (Table 2). The FPLD group had reduced thigh skinfold thickness (19.5 mm vs 48.2 mm, *p* = 0.008) with a correspondingly increased subscapular to thigh skinfold ratio (2.23 vs 0.84, *p* = 0.004), and suprailiac to thigh skinfold ratio (1.91 vs 0.77, *p* = 0.004). There was no significant difference for skinfold measurements of biceps, triceps, scapular, abdominal, calf and sum of 7-site skinfolds. The subscapular to calf ratio and suprailiac to calf ratio trended higher in the FPLD group compared with controls but did not reach statistical significance.

The comparison of distribution of adipose tissue using bioelectrical impedance analysis, DXA and MRI are shown in Table 3. The body fat percentage, fat mass and muscle mass between the two groups were well-matched as measured by bioelectrical impedance and DXA.

**Table 3:** Adipose tissue distribution in females with familial partial lipodystrophy (FPLD) compared with females without partial lipodystrophy, using bioelectrical impedance analysis, DXA and MRI.

	FPLD (*n* = 8)	Controls (*n* = 4)	*p* value
***Bioelectrical Impedance Analysis (Tanita)***			
Fat %	43.7 ± 4.6	45.9 ± 8.6	0.461
Fat mass (kg)	33.3 ± 5.4	38.1 ± 12.9	0.570
Muscle mass (kg)	40.3 ± 5.7	40.5 ± 1.9	0.683
***DXA***			
Total body fat %	41.4 ± 4.0	45.3 ± 4.1	0.234
Total lean mass/height^2^	17.3 ± 1.4	17.2 ± 2.6	0.683
Body fat mass index	13.0 ± 2.4	15.3 ± 4.0	0.275
Head fat %	23.2 ± 0.9	22.6 ± 0.6	0.141
Trunk fat %	45.8 ± 4.3	45.2 ± 4.6	1.000
Right arm fat %	50.4 ± 4.4	51.8 ± 7.3	0.461
Right leg fat %	33.3 ± 6.0	47.6 ± 2.8	0.004
Abdominal VAT volume (cm^3^)	1093 ± 203	1076 ± 394	0.933
Android to gynoid ratio	1.27 ± 0.20	0.99 ± 0.05	0.008
Fat % trunk to fat % legs ratio	1.43 ± 0.26	0.95 ± 0.05	0.004
Trunk to limb fat mass ratio	1.72 ± 0.32	1.01 ± 0.08	0.004
***MRI***			
*Abdomen*			
Abdominal SAT volume (cc)	4835 ± 1309	4916 ± 1830	1.00
Abdominal superficial SAT volume (cc)	3010 ± 561	3011 ± 1031	0.933
Abdominal deep SAT volume (cc)	1824 ± 793	1904 ± 834	0.808
Abdominal VAT volume (cc)	3048 ± 690	2496 ± 1114	0.461
Intraperitoneal adipose tissue (cc)	1909 ± 489	1588 ± 792	0.569
Retroperitoneal adipose tissue (cc)	1004 ± 229	778 ± 329	0.367
Paraspinal adipose tissue (cc)	130 ± 44	128 ± 18	0.808
Total abdominal adipose tissue (cc)	7880 ± 1668	7410 ± 2842	0.808
*Legs*			
Thigh muscle volume (cc)	2335 ± 479	2276 ± 457	0.808
Thigh SAT (cc)	2350 ± 750	4921 ± 1243	0.004
Thigh IMAT (cc)	1307 ± 281	1346 ± 296	0.933
Calf muscle volume (cc)	1087 ± 132	1052 ± 106	0.933
Calf SAT (cc)	442 ± 155	927 ± 292	0.008
Calf IMAT (cc)	179 ± 53	171 ± 80	0.570
Gluteal SAT (mm)	23.0 ± 6.1	42.0 ± 5.6	0.004
*Ratios*			
Abdominal VAT to Abdominal SAT ratio	0.65 ± 0.16	0.50 ± 0.15	0.214
Abdominal VAT to Thigh SAT ratio	1.48 ± 0.75	0.49 ± 0.15	0.004
Abdominal VAT to Calf SAT ratio	8.10 ± 4.54	2.64 ± 0.93	0.004
Abdominal SAT to Thigh SAT ratio	2.29 ± 1.01	0.98 ± 0.12	0.008
Abdominal SAT to Calf SAT ratio	12.2 ± 4.9	5.2 ± 0.4	0.016
MRI PDFF*			
Liver PDFF (%)	18.7 ± 11.9	12.2 ± 8.6	0.570
Pancreas PDFF (%)	5.0 ± 4.3	3.7 ± 2.3	0.808
Thigh muscle PDFF (%)	8.4 ± 3.3	6.8 ± 1.3	0.570
Calf muscle PDFF (%)	8.4 ± 2.3	6.8 ± 2.3	0.283
Intra-and Extra myocellular lipids**			
IMCL (%)	2.32 ± 1.40	1.74 ± 1.53	0.461
EMCL (%)	2.70 ± 1.12	2.22 ± 0.91	0.434
IMCL to EMCL ratio	1.09 ± 0.86	1.05 ± 1.18	0.683

Values represented as mean ± standard deviation or number (%).

Body Fat Mass Index (fat mass/height2), *VAT* visceral adipose tissue, *SAT* abdominal subcutaneous adipose tissue.

*IMCL* Intramyocellular lipids (%), *EMCL* Extramyocellular lipids, *IMAT* intramuscular adipose tissue, *PDFF* proton density fat fraction.

*Fat % trunk to fat % legs ratio* percentage of fat in trunk divided by percentage of fat in legs.

*Liver, pancreas, and skeletal muscular fat (thigh and calf) were expressed as proton density fat fraction.

**IMCL and EMCL were expressed as a ratio with respect to water.

**Fig. 1:**
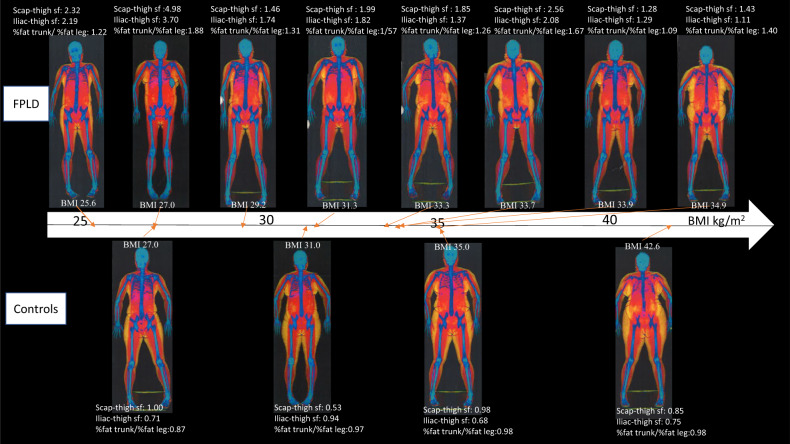
Fat shadows derived from DXA scans of patients with FPLD type 1 and control participants with their skinfold readings, across BMI are shown. A decreased fat shadow was observed in the gluteofemoral and thigh regions in patients with FPLD. Scapular to thigh skinfold ratio (‘scap-thigh sf’), suprailiac to thigh skinfold ratio (‘iliac-thigh sf’) and the DXA-derived variable % fat of trunk to % fat of leg ratio are shown.

Based on DXA, the FPLD group had reduced leg fat compared with the control group (33.3% vs 47.6%, *p* = 0.004). As the trunk fat percentage was similar in both groups, there was a corresponding increased ratio of trunk to leg fat percentage (1.43 vs 0.95, *p* = 0.004), ratio of trunk to limb fat mass (1.72 vs 1.01, *p* = 0.004) and ratio of android to gynoid (1.21 vs 0.98, *p* = 0.008) in the FPLD group. The fat shadows derived from DXA scans in the FPLD group showed that with increasing BMI, there was symmetrically increased subcutaneous fat depots over upper body, neck, and the abdomen, but with reduced subcutaneous fat depot at the legs (thighs and calf) (Fig. 1). This was unlike the control subjects where there was a general increase in subcutaneous fat deposition including all extremities with increasing BMI (Fig. 1).

**Fig. 2:**
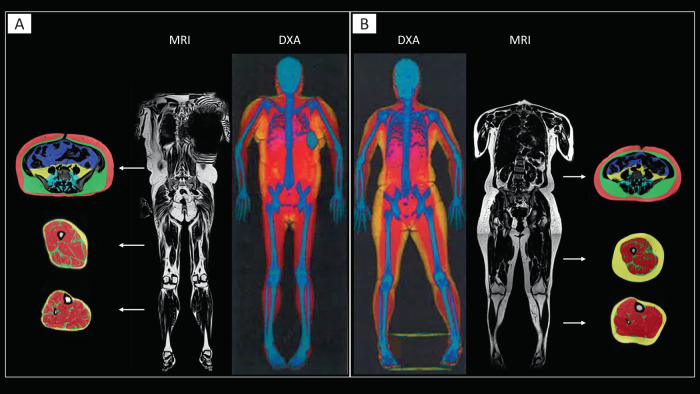
MRI and DXA images of a female patient with partial lipodystrophy and a control with identical BMI. Representative MRI and DXA images from a female patient **A** with FPLD and **B** a control subject who have identical BMI (27 kg/m^2^). The FPLD patient **A** showed markedly different body fat distribution with higher abdominal deep subcutaneous adipose tissue (green), superficial subcutaneous adipose tissue (red), intraperitoneal adipose tissue (blue), retroperitoneal adipose tissue (yellow), and paraspinal adipose tissue (cyan), and selective loss of SAT in the thigh and calf compared to the control subject **B**. The artifact in the MRI image **A** is due to an implantable cardioverter defibrillator.

Based on MRI, the FPLD group had a decreased thigh SAT volume (*p* = 0.004) and calf SAT volume (*p* = 0.008) compared to controls, while there was no statistical difference in the thigh muscle and calf muscle volumes. Correspondingly, the abdominal SAT to femoral and calf SAT ratio were both increased (*p* < 0.01). The gluteal fat thickness was significantly lower in the FPLD group compared with controls (23 vs 42 mm, *p* = 0.004). Figure 2 illustrates two female individuals with identical BMI of 27 kg/m^2^ and height 1.56 m, but with markedly different body fat distribution in DXA and MRI. There was an increased abdominal SAT and VAT volume, but markedly reduced gluteofemoral SAT (thigh and calf shown in Fig. 2) in patient A (Patient #2 on Table 1) compared with the BMI and height-matched control. Patient A had premature onset of heart failure with reduced ejection fraction, DM nephropathy with reduced GFR of 50 ml/min, albuminuria, and severe diabetes mellitus on multiple insulin injections per day.

### Intra-abdominal and intra-muscular fat

Hepatic, pancreatic, and muscular (thigh and calf) PDFF measured by MRI in the cases and controls are shown in Table 3. Both the mean hepatic PDFF (18.7 vs 12.2) and pancreatic PDFF (5.0 vs 3.7) appeared higher in the FPLD group compared with the control group, but were not statistically significant. All patients in the FPLD group and controls had hepatic steatosis as defined by MRI-PDFF ≥ 5% [23]. The thigh and calf muscles’ fat and IMAT were not different between the two groups. The intramyocellular and extramyocellular lipid percentages trended slightly higher in the FPLD group compared with the control group but were not statistically significant.

## Discussion

The potential important findings from this study were as follows. First, there was significantly reduced subcutaneous adipose tissue but not muscular volume in the legs of patients with FPLD1 compared with controls, demonstrated by DXA, MRI, and skinfold thickness. This refutes the possible misconception that the thin legs of patients with FPLD due to muscle wasting. Second, we showed that a low skinfold of the anterior thigh, skinfold ratios (subscapular/thigh, and suprailiac/calf ratio), and DXA-derived ratio of trunk to leg fat percentage were potentially clinically helpful tools to differentiate FPLD from controls. Third, patients with polygenic type of FPLD can have severe cardiometabolic phenotype.

Approximately 85% of total adipose tissue mass in healthy individuals is in the subcutaneous compartment, reflecting its importance as a major site of energy storage [24]. In lipodystrophies, the loss of subcutaneous white adipose tissue is associated with free fatty acid metabolism dysregulated in visceral adipose tissue and increased deposition in liver, pancreas and muscle, and endothelial dysfunction [4, 25–27]. Independent of VAT or upper body fat depot, studies have shown that increased lower body fat particularly gluteofemoral fat, is independently associated with reduced cardiovascular risk, and conversely, reduced lower body fat is associated with increased cardiovascular risk [4, 21, 26, 28]. This may be because gluteofemoral subcutaneous white adipose tissue is relatively more insulin sensitive and has a lower lipolytic rate, whereas VAT and abdominal SAT are more insulin resistant and has a higher lipolytic rate [4]. Unlike the lipodystrophy of the lower leg fat depot, the localised or partial lipodystrophy of the arms or upper trunk is not typically associated with metabolic disease [4]. The theory of lack of expandability of SAT in the legs in partial lipodystrophy, is in keeping with our observation that there is a relative decrease in fat and adipose tissue in the legs across the BMI range.

Alteration of adipokine secretion from adipose tissue in lipodystrophies, particularly of leptin and adiponectin also contribute to the metabolic complications of lipodystrophies, more so for generalised than partial lipodystrophies [4]. In our study, leptin concentrations were in the lower half of the reference interval but were not critically low. In lipodystrophies, low levels of adiponectin are related to the loss of adipose tissue, especially leg fat [29], and increased insulin resistance [4, 26].

We found that subscapular-to-thigh ratio and suprailiac-to-thigh ratio were significantly higher in FPLD1 patients compared with controls, suggesting that these skinfold measurements could be useful screening and diagnostic tools. In a Spanish study of 98 patients with FPLD1 compared with 60 controls, the subscapular to calf ratio (KöB index) >3.477 was highly sensitive (89%) and specific (84%) for diagnosis of FPLD1 [30]. In this study, the mean ± SD skinfold for thigh and calf were 20.5 ± 10.7 mm and 6.3 ± 4.4 mm respectively, and mean DXA-lower limb fat 33.3% [30]. Unlike their study, we found that subscapular to calf ratio was less discriminatory, noting that our study is limited by small sample size. In a study of 50 female patients with FPLD2 caused by genetic mutations in *LMNA*, the DXA-derived measures of lower limb fat percentage and the lower limb fat to truncal fat ratio were mostly ≤ 1^st^ percentiles of NHANES [31]. The authors suggested that a low leg fat percentage and a low thigh skinfold of <22 mm (corresponding to <10^th^ percentile of adult females in USA) [1, 31], and were useful markers to increase the diagnostic suspicion of FPLD2 [31].

We showed that the DXA-derived variable ratio of trunk to legs fat percentage was significantly increased in the 96–99^th^ percentile, as reported by Hologic software which uses the normative reference from the NHANES dataset [32]. Using a Singapore reference population dataset, the android to gynoid fat ratio of our case series also corresponded to >99^th^ percentile for the age and sex-specific percentiles, while the trunk/limb fat mass ratio corresponded to >97^th^ percentiles in all patients except one [33]. Our study findings are supported by other small studies of FPLD which reported that ratio of trunk to leg fat percentage of >1.5 or ratio between trunk and lower limbs fat mass (called fat mass ratio) of >1.2, to be potentially sensitive markers [5, 10]. Larger studies in the Asian population are needed to confirm the best thresholds for thigh skinfold, the ratio of trunk to leg fat percentage on DXA, for clinical use, in different genders, age groups, and ethnicities. Our pilot study suggests that using both thigh skinfold ≤ 3 cm and ratio of trunk to leg fat percentage of ≥1.2 on DXA in young women (age 25–45) with BMI around 25–35 kg/m^2^, in the presence of a positive family history, are potential useful clinical markers to help clinicians identify and diagnose patients with FPLD. Thus, facilitating the identification of these high-risk individuals who urgently requires intensification of management to mitigate their cardiometabolic risk.

While FPLD type 1 is polygenic, the other types of FPLD are categorized by the presence of a pathogenic, usually dominant, gene variant. For example, FPLD2 is caused by variants in *LMNA* and FPLD3 is caused by variants in *PPARG*. Therefore relying solely on a physician’s clinical acumen for detecting signs of symmetrical distal lipoatrophy makes a confident diagnosis of FPLD challenging, particularly in individuals who are lean or male gender [1, 9, 34]. Subtyping FPLD without genetic testing is also challenging because the genotype-phenotype correlation differentiating the subtypes of FPLD is not clinically apparent; clinical features of distal lipoatrophy could be related to any of the monogenic forms of FPLD including *LMNA*, *PPARG*, *PLIN1*, *CIDEC*, *LIPE*, *AKT2*, and *ADRA2A*, or caused by polygenic influences (i.e. FPLD1) [4]. The utility of polygenic risk scores of lipodystrophy in clinical practice remains to be investigated. Further studies are required to investigate the role of ethnicity-specific polygenic risk scores.

The strength of our study includes extensive imaging (MRI and DXA) and comprehensive measurements of body fat and muscle mass distribution. The main limitation of our study was the small sample size and consisting only of women. Diagnosis of FPLD in males is challenging due to the android nature of the body composition of the male gender. However, women with FPLD appear to have greater risk of metabolic dysfunction than males, presumably because women normally have increased fat stores generally and at the lower limbs [4, 8]. We did not study pericardial or intramyocardial fat, or have comparison group of patients with monogenic FPLD. Although the use of skinfolds has been postulated to be useful in our study and in others, skinfold measurement carries inter-and intra-variability error. While our study was underpowered to conclude that ectopic deposition of pancreatic fat and liver fat were increased between groups as reported by another small study [25], the mean levels of liver fat appeared much higher in the FPLD group (but not statistically significant). Interestingly, the mean volumes of intramuscular, retroperitoneal, and paraspinal adipose tissues were very similar between the groups, however, larger studies are needed to study this. Our pilot study is useful to guide future larger studies to improve the detection and diagnosis of FPLD.

## Conclusion

Patients with polygenic FPLD have significant loss of adipose tissue in lower limbs and early onset of diabetes and metabolic complications. Early diagnosis is possible with careful physical examination of the lower limbs (gluteus, thigh, calf) and early intensification of metabolic management is critical. Reduced thigh skinfolds, and an increased ratio of trunk to legs fat percentage on DXA are potentially clinically useful markers to identify FPLD. Larger and more detailed analyses are required to confirm the clinical utility of these markers. Long term surveillance of this patients with FPLD, such as facilitated by registries may provide further insight into understanding of FPLD.

### Supplementary information

Supplementary Tables 1 and 2

## Data Availability

The datasets generated during and/or analyzed in the current study are available from the corresponding author upon reasonable request.
