# Supplementary material for: Clinical and imaging features of women with polygenic partial lipodystrophy: a case series
Source: Nutr Diabetes. 2024 Feb 6;14:3. doi: 10.1038/s41387-024-00260-y (PMC10847407; doi:10.1038/s41387-024-00260-y)
Supplement: Supplementary file 1 — Supplementary Tables 1 and 2 [file 41387_2024_260_MOESM1_ESM.pdf]

Supplementary Tables for Manuscript titled Clinical and Imaging Features of Women with Polygenic Partial Lipodystrophy: A Case Series

Supplementary Table 1: Detailed descriptions of skinfold measurement, DXA, genetics and MRI techniques used.

Dual-energy X-ray absorptiometry (DXA)

DXA scans were performed using Hologic QDR 4500A, fan-beam densitometer (Hologic, Inc., Bedford, Massachusetts, software version 8.21) to measure whole body composition. Radiation exposure from each whole-body scan was less than 10 μSv. Body composition measures included fat of total body, head, trunk, arms and legs, abdominal visceral adipose tissue (VAT), android and gynoid fat masses and ratio of trunk to leg fat percentage. The variable ratio of trunk to leg fat percentage is automatically derived by Hologic software which reported the variable as '%fat trunk to % fat of legs ratio'. The abdominal VAT measured on DXA was defined by the patient's abdominal cavity between pelvis and the rib cage, corresponding to the L4-L5 level. Android and gynoid regions were defined by the Hologic APEX software. The android region was defined as the lower trunk area bounded by two lines: the pelvic horizontal cut line on its lower side, and a line automatically placed above the pelvic line. The upper gynoid line was placed 1.5 times the height of the android region below the pelvic line and the lower gynoid line was placed such that the distance between the two gynoid lines was twice the height of the android region. All these lines were automatically placed by Hologic software. The precision estimate (%CV) for fat mass using DXA measurement ranges from 1-2%. Long term precision QC monitoring on phantoms for three years on the Hologic densitometer was 0.01%.

Genetics

Whole genome sequencing was performed by Beijing Genomics Institute (BGI). In brief, genomic DNA was fragmented by sonication and size selected for 250bp. Adapters were ligated to both ends of the end repaired/dA tailed DNA fragments, amplified by ligation-mediated PCR then single strand separation and cyclization performed. Rolling circle amplification was performed to produce DNA Nanoballs, which were sequenced on the BGISEQ-500 platform. Reads were aligned to the human reference genome GRCh37/hg19. SNPs and InDels were detected using GATK HaplotypeCaller (v3.3.0).

Abdominal fat

MRI was performed using the Siemens Prisma 3T MR scanner. Abdominal MRI was performed and axial image slices with 3 mm thickness, 0.6 mm interslice gap, and in-plane resolution of 1.3 × 1.3 mm were acquired for each subject using a two-point Dixon fat-water imaging sequence (TR = 4.09 ms, TE1 = 1.23 ms, TE2 = 2.46 ms) and body matrix coil. The images were acquired from the abdominal region between L1 and L5 vertebrae during a breath hold of 18s. A deep learning based automatic segmentation algorithm followed by manual editing was used to segment and quantify the deep subcutaneous (DSAT), superficial subcutaneous (SSAT), intraperitoneal (IPAT), retroperitoneal (RPAT), and paraspinal adipose tissue (PSAT) compartments [19].

Liver and pancreatic fat

The liver and pancreatic fat were determined using multi echo Dixon fat-water imaging sequence (TR = 15 ms, 8 echoes, TE1 = 1.23 ms, ΔTE = 1.24 ms) and body matrix coil. Multiple regions of interest (ROIs) were selected within the liver carefully excluding the blood vessels and boundaries in the fat fraction image. The liver fat was quantified as the mean proton density fat fraction within the ROIs. To quantify pancreatic fat, multiple ROIs were drawn within the head, body, and tail regions of the pancreas. The pancreatic fat within the head and body was determined by computing the mean proton density fat fraction within ROIs selected in the head and body of the pancreas. Similarly, the pancreatic fat in the tail region was quantified as the mean proton density fat fraction within ROIs in the pancreatic tail.

Fat and muscle in the thigh and calf

MRI of the calf and thigh were acquired using proton density turbo spin echo sequence (TR = 3200 ms, TE = 52 ms, slice thickness = 5 mm, in-plane resolution = 0.9 × 0.9 mm) covering the region from the top of femoral head to the patella for the thigh and from patella to the bottom of the fibula for the calf in four slabs. A custom developed

algorithm followed by manual editing was used to segment the subcutaneous fat, muscle, and inter- and intramuscular adipose tissue (IMAT) in the thigh and calf.

Calf and thigh muscle fat fraction

The thigh and calf muscle proton density fat fraction were determined using (TR = 15 ms, 10 echoes, TE1 = 1.23 ms, ΔTE = 1.24 ms). ROIs were drawn in four representative proton density fat fraction images in the thigh region covering the vastus (lateralis, intermedius, and medialis) and femoris muscles and the mean proton density fat fraction was computed. Similarly, ROIs were drawn in calf region covering the gastrocnemius (lateralis and medialis) and soleus muscles to compute calf muscle proton density fat fraction.

Intra- and extramyocellular lipids (IMCL and EMCL)

Fat in the skeletal muscle was determined using magnetic resonance spectroscopy. The muscle spectrum was obtained from a $2 \times 2 \times 2$ cm^3 voxel within the soleus muscle using a point resolved spectroscopy sequence (TR = 2000 ms, TE = 30 ms). The spectrum was quantified using LCModel[32]and the amount of intramyocellular (IMCL) and extramyocellular (IMCL) lipids was calculated and expressed as a ratio with respect to water and corrected for T_2 losses (1,2).

References:

[1] Alexandra Kautzky-Willer et al. Increased Intramyocellular Lipid Concentration Identifies Impaired Glucose Metabolism in Women With Previous Gestational Diabetes. Diabetes February 2003 vol. 52 no. 2 244-251.

[2] Krššák M, Lindeboom L, Schrauwen-Hinderling V, Szczepaniak LS, Derave W, Lundbom J, Befroy D, Schick F, Makhan J, Kreis R, Boesch C. Proton magnetic resonance spectroscopy in skeletal muscle: Experts' consensus recommendations. NMR Biomed. 2021 May;34(5):e4266.

Supplementary table 2: Heterozygous rare nonsynonymous variants in lipodystrophy and related genes of patients with familial partial lipodystrophy.

Patient with FPL	Reference seq.	Gene	Nucleotide	Protein	dbSNP	gnomAD E Asian MAF
No.7	NM_000553.4<br>NM_000681.4	WRN<br>ADRA2A	c.1579T>G<br>c.752C>G	p.Phe527Val<br>p.Pro251Arg	-<br>rs375697365	-<br>32/7478
No.6	-					
No.8	NM_001374769.1<br>NM_000138.4	SIM1<br>FBN1	c.1793C>G<br>c.6987C>G	p.Ala598Gly<br>p.Asp2329Glu	rs199641634<br>rs363831	62/18854<br>159/18830
No.3	NM_001873.4<br>NM_001287248.2	CPE<br>BLM	c.459C>G<br>c.1992A>G	p.Ile153Met<br>p.Ile664Met	rs756934559<br>rs576199850	73/18778<br>52/18594
No.2	-					
No.4	-					
No.1	NM_032737.4	LMNB2	c.1831A>G	p.Arg611Gly	rs1269271046	0/17242
No.5	NM_001374769.1<br>NM_012232.6	SIM1<br>CAVIN1	c.1525T>A<br>c.22A>G	p.Tyr509Asn<br>p.Ile8Val	rs780931884<br>rs757445859	2/16434<br>2/15040

List of genes investigated:

ABCC8	EPHX1	MANF	SH2B1
ADRA2A	FBN1	MC3R	SIM1
AGPAT2	FGFR3	MC4R	SLC16A1
AGPS	GATA4	MFN2	SLC29A3
AKT2	GATA6	NEUROD1	TBC1D4
ALMS1	GCK	NPY	TRMT10A
ANGPTL3	GCKR	NR0B2	TUB
ANGPTL4	GIGYF1	NSMCE2	UCP2
APOA5	GNAS	NTRK2	UCP3
APOC2	GPD1	PAX6	WFS1
APOC3	GPIHBP1	PCBD1	WRN
APPL1	HNF1A	PCSK1	ZBTB20
BLM	HNF1B	PCYT1A	ZFP57
BSCL2	HNF4A	PDX1	ZMPSTE24
CAV1	INS	PIK3R1	
CAVIN1	INSR	PIK3R1	
CEL	KCNJ11	PLIN1	
CEP19	KSR2	PNPLA2	
CIDEC	LEP	POC1A	
CISD2	LEPR	POLD1	
CP	LIPE	POLR3A	
CPE	LMF1	POMC	
CREB3L3	LMNA	PPARG	
DCAF17	LMNB2	PPP1R15B	
DNAJC3	LPIN1	PPP1R3A	
DUT	LPL	PSMB8	
DYRK1B	MAFA	RFX6	
